# Periodontal status of textile versus biscuit industrial workers in India: A comparative study

**DOI:** 10.6026/973206300220669

**Published:** 2026-02-28

**Authors:** Vipul Yadav, Aditi Verma, Jagreeti Kayasth, Naveen Arumugam, Shreya Bhukal, Geetanjali Yadav

**Affiliations:** 1 Department of Public Health Dentistry, Post Graduate Institute of Dental Sciences, Rohtak, India; 2 Department of Public Health Dentistry, Faculty of Dentistry, Jamia Millia Islamia, New Delhi, India; 3 Department of Cardiology, All India Institute of Dental Sciences, New Delhi, India; 4 Department of Zoology, Zakir Hussain Delhi College, Delhi University, India

**Keywords:** Periodontal health, biscuit industrial workers, textile industrial workers

## Abstract

Occupational exposures threaten periodontal health among industrial workers through heightened infection susceptibility. Therefore, it is of interest to compare periodontal status using Community Periodontal Index (CPI) and Loss of Attachment (LOA) indices in randomly selected biscuit (n=2 factories) and textile (n=1 factory) workers from Delhi. Biscuit workers showed higher healthy/low-score sextants (bleeding, calculus) versus textile workers (p<0.001). Textile workers had significantly more deep pockets (4-5 mm, >6 mm) and excluded sextants (p<0.001). Textile work adversely affects periodontal health more than biscuit manufacturing, informing targeted occupational oral health screening and interventions.

## Background:

Health is often been ignored by the community, in spite of continuous effort been made towards health promotion, worldwide [1]. Oral health is the mirror for the general health and an important asset for any individual. Health has multidimensional component and multiple determinants, among which occupational health is major determinants of health. Health is often being noticed to be neglected among certain working class who often spend their time in travelling from one place to another, without regular food, rest, recreation and even sleep. These people often work in abnormal climatic conditions with frequent change in their day and night shifts leading to disturbances in their circadian rhythm, their lifestyle which is compounded by delays and breakdowns [2]. Oral cavity is a gateway for many diseases as it presents with several unique features which make it especially prone to occupational diseases. An individual life-cycle is composed of multiple oral diseases and dysfunctional oral conditions which has a profound impact on the quality of life. Oral diseases are often considered to be expensive in terms of financial resources, tooth loss, pain and fear, trauma and anxiety and time lost from work [3]. In developing countries, such as India, risk of diseases and deaths are often associated with poor nutrition, personal and environmental hygiene, customs and cultural patterns, where traditional lifestyles still persist. These risk factors play an important role in aetiology of adult periodontal disease [4]. In country like India, millions of people daily worked in a dusty environment. The frequently reported symptoms in textile mill workers are cough, byssinosis, chest tightness, bronchial asthma, fatigue and also carcinoma of lung, stomach and colon.

Chronic respiratory diseases are due to cotton dust inhalation and varicose veins to low backache due to working postures. However, with respect to oral cavity, the commonly reported symptoms in textile mill workers are inflammation of gums (gingivitis), calculus and pocket formation [5]. Ill effects on the health are often associated with almost all occupational diseases with studies showing association between occupational exposure and higher incidence of oral diseases. Some examples are the relation between oral diseases and working environment in cement industries, bidi factory and food industries [6, 7-8]. As factory workers constitute a well - defined population group, knowledge of factors affecting their oral health in a work place allows oral health promotional measures to be appropriately targeted. Studies conducted on them also helps in planning of preventive programmes for the prevention of oral diseases and promotion of oral health in industrial health care system. Surprisingly, in developing countries, very little is known about how various occupations have its effects on oral health. Therefore, it is of interest to assess and compare the periodontal health status of biscuit and textile industrial workers in Delhi.

## Material and Methods:

The present study was a comparative cross-sectional study to evaluate and compare the periodontal health of biscuit and textile industrial workers of Delhi. There were total 3 biscuit and 2 textile industries in Delhi with a total population of 2010 production line workers. Out of total workers, 846 production line workers were in Biscuit and 1164 production line workers were employed in textile industries. With the national prevalence of periodontal diseases of 60% at 95% CI and 5% significance level, a sample size of 1100 workers were obtained. From the sample obtained, 580 were selected randomly from textile industry and 520 from biscuit industries. To fulfil this sample size, two biscuit industries and one textile industry were selected which formed the sampling frame. The industries were selected on the basis of Probability proportional to enrolment size (PPE) i.e., industries with higher number of workers are more likely to be selected than industries with a smaller number of workers. The inclusion criteria included study subjects aged 21 years and above with working experience more than 2 years. The exclusion criteria included subjects, who were physically challenged, medically compromised and contract workers (those workers who were recruited for a short period of time when production rate is on higher side). Written permission to conduct the oral health examination was obtained from the Managing director of all the factories by explaining the aim and objectives of study. Ethical clearance was sought from the Institutional Ethical Committee. Informed consent was obtained from workers participating in the study well before the clinical examination. The examination was conducted at respective centre (Biscuit and textile industries) in a separate well illuminated and ventilated room.

A self- designed pretested proforma was used to collect demographic details along with clinical findings. The proforma included questions regarding personal data, education level, duration of employment, oral hygiene aids and practices, frequency and duration of tobacco and alcohol consumption. A pilot study was conducted among 30 production line workers to check the feasibility of the study and Proforma. A survey was systematically scheduled for a period of 6 months. Type III clinical examination and recording of the proforma was carried out by the principal investigator. To determine intra-examiner variability, the periodontal examination of 10 randomly selected subjects was repeated on different dates. The kappa coefficient value for intra- examiner reliability with respect to different variables for the CPI and LOA ranged from 0.82-0.92. Community periodontal index (CPI) and loss of attachment index (WHO 2013 criteria) was sued to assess the periodontal health among both biscuit and textile industrial workers [8]. All the statistical tests were performed using Statistical Package for Social Sciences (SPSS version 17.0) Mean values were compared using student t-test and ANOVA (P<0.05). Multiple logistic regression analysis was used to assess the multiple independent variables with dependant variables among biscuit and textile industrial workers.

## Results:

Out of total 1100 subjects enrolled in the study, 520 (47.3%) were from biscuit industry while remaining 580 (52.7%) were from textile industry. The age of biscuit industry workers ranged from 20 to 48 years with a mean age of 29.12±6.11 years. Age of textile industry workers ranged from 21 to 59 years with a mean age of 32.27±8.56 years (Table 1). Among biscuit industry workers, majority (n=417; 80.2%) used to clean their teeth with brush, followed by those using finger (n=81; 15.6%). Only 22 (4.2%) workers used Datoon to clean their teeth. However, among textile industry workers, maximum number of workers (n=253; 43.6%) used to clean their teeth with brush, a substantial number used finger (n=199; 34.3%) and Datoon (n=114; 19.7%), while 14 (2.4%) did not practice cleaning of teeth. Only 6.7% of biscuit industry workers had habit of tobacco use while 19 (3.7%) workers accepted having the habit of alcohol use. In Textile industry, both tobacco and alcohol use were much higher as compared to that in biscuit industry. A total of 170 (29.3%) workers had habit of tobacco use while a total of 118 (20.3%) had habit of alcohol use. On comparing the two groups, a statistically significant difference was observed for both tobacco as well as alcohol use (p<0.001) (Table 1).

Irrespective of any adverse habits (tobacco or alcohol), mean CPI scores were higher in textile industry workers compared to biscuit industry workers (p<0.001). Within group comparisons for different habits revealed no significant differences in biscuit industry workers, however, the differences in textile industry were statistically significant (p<0.001) showing mean CPI scores to be significantly lower among those with no habit or habit of alcohol only as compared to those having habit of tobacco or habit of tobacco in combination with alcohol. Similarly, irrespective of the adverse habit, mean LOA scores was higher in textile industry workers compared to biscuit workers, however, the difference was statistically non- significant for those with habit of alcohol only (p=0.587). Within group comparison for effect of different habits on mean LOA did not reveal a significant difference among biscuit industry workers (p=0.265). However, differences in textile industry were statistically significant showing mean LOA scores to be significantly lower among those with no habit or habit of alcohol only as compared to those having habit of tobacco or habit of tobacco in combination with alcohol (p<0.001) (Table 2). Multiple Regression analysis showed association of CPI (>3) with duration of employment, type of education and type of industry. When adjusted for duration of employment and education, among the type of industry, textile industrial workers (2.36) showed a higher association with periodontal status than the biscuit industry (0.13). When adjusted for type of industry and education, with an increase in duration of employment there was an increase in the adjusted odds ratio for periodontal status. When adjusted for type of industry, duration of employment, for the education, those workers who were educated till primary were found to have higher CPI score than workers educated till intermediate and high school. This suggest that all variables (education, duration of employment, type of industry) independently had an association with CPI >3 (p<0.001) (Table 3).

**Figure 1:**
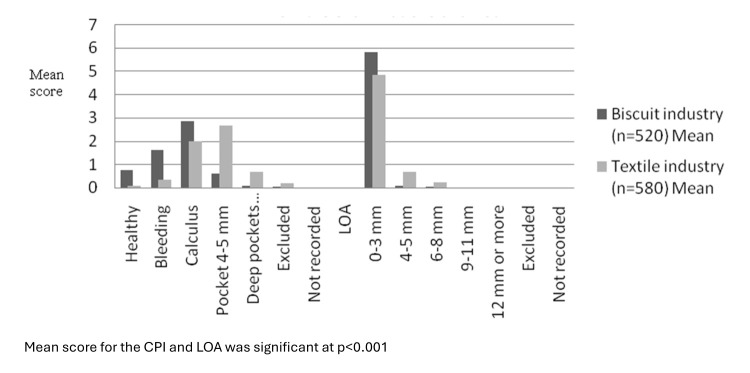
Mean sextants with healthy periodontal tissue, bleeding or higher score, calculus or higher score, deep pockets along with mean number of sextants with loss of attachment and mean number of sextants excluded from the examinations and not recorded.

Similarly for the LOA, multiple regression analysis showed the association of duration of employment, type of education and type of industry with LOA. When adjusted for duration of employment and education, among the type of industry, textile industrial workers (2.60) were found to have higher association with loss of attachment than the biscuit industry (0.11). When adjusted for type of industry and education, with an increase in duration of employment there was a decrease in the adjusted odds ratio for loss of attachment. When adjusted for type of industry, duration of employment, for the education, those workers who were educated till primary were found to have higher loss of attachment than workers educated till intermediate and high school. This suggest that all the variables (education, duration of employment and type of industry) independently had an association with loss of attachment (p<0.001) (Table 4). Mean number of sextants with code 0, 1 and 2 score (healthy, bleeding, calculus) were higher among biscuit workers compared to textile workers (p<0.001). However mean sextants with CPI code of periodontal pocket 4-6 mm and pocket >6 mm was higher in textile industrial workers (p<0.001). However, mean sextants with LOA 0-3 mm was higher among biscuit workers compared to textile workers (p<0.001). For higher LOA (4-5 mm and 6-8 mm) mean number of sextants was significantly higher in textile industry as compared to biscuit industry (p<0.05) (Figure 1).

## Discussion:

Health has evolved over the centuries as a concept from an individual concern to a world-wide social goal and encompasses the whole quality of life. The health status of an individual, a community or a nation is determined by the interplay and integration of two ecological universes- "the internal environment of man himself and the external environment, which surround him" [9]. Work place is really worshipped where there are interactions between people and the working environment along with performing job. Industrial workers constitute a well-defined population but due to their poor life styles, huge chunks of this population suffer from oral diseases. In developing countries millions of people daily work in a dusty or polluted environment, the biscuit and textile industries are one of the industries that produce sugar dust and cotton dust respectively, along with fumes during manufacturing processes, which contribute to the poor health including oral health. The individual, community lifestyle or ways of living is being determined by environment to which individuals have been exposed throughout their lives from birth to present time have comprised the individual and community life styles, or ways of living. Factory employees constitute well defined population groups and although not representing nationwide samples, such groups are often readily available and therefore have several practical advantages in epidemiologic studies [10]. The present study was a cross- sectional study to assess the periodontal health status of biscuit and textile industrial workers of Delhi. Out of total 1100 subjects enrolled in study, 520 were from biscuit and 580 were from textile industries. The overall mean age of biscuit industrial workers is 29.12 ±6.11 while that of textile workers is 32.27±8.56. In both the groups no female subjects were present. The result showed the mean number of sextants with score >3 of periodontal disease and loss of attachment was higher in the textile industry as compared to biscuit industrial workers which was similar to study done by authors Backanek *et al.* [11]. This may be attributed to oral hygiene aids and frequency of tooth cleaning which was better among the subjects in the biscuit industrial workers than the textile workers. The subjects in the upper class (biscuit workers) will have a better knowledge on the usefulness of oral hygiene aids and oral hygiene practices in the prevention of oral diseases which may be lacking among the lower classes (textile workers). Besides, the lack of affordability to buy the oral hygiene aids may prompt the people in the lower classes to look out for cheaper alternates in the form of charcoal, mud and so on along with finger that is detrimental to the oral health. These findings were similar to study done by Chandra Shekar *et al.* [12] in Mysore who also reported the same findings which is in support with our study.

The mean sextant with bleeding on probing and calculus was higher (among biscuit industrial workers than the textile industrial workers which may be greatly attributed to the effect of the work environment (sugar dust) which resulted in greater gingivitis, which was similar to findings reported by Masalin *et al.* [13] while the mean sextants with periodontal pocket (4mm and ≥ 4mm) and loss of attachment was higher (2.67±1.39) among textile industrial workers as most of the workers in textile industry are illiterate, belongs to higher age group than biscuit industrial workers, uses finger and datoon (cleaning twig) as an oral hygiene aids for cleaning teeth and also had greater tobacco consumption resulting in higher periodontitis than biscuit industrial workers. The findings were consistent with the survey result from Finnish industrial population which concluded that there was progression of periodontal disease with advancing age which supports our findings [14]. The continuous increase of bone loss with increasing age is consistent with the results from previous study done by Macdonald *et al.* [15]. The study done by Vianna *et al.* reported that non-occupational factors like education, smoking and socioeconomic factors appeared to be more relevant for periodontal diseases. Smoking is considered as one of the risk factor for periodontal disease, negative association with gingival bleeding and other inflammatory signs have been reported due to vasoconstriction caused by nicotine [16]. It is well known fact that workers in the textile industries are paid less and are in continuous direct exposure to fibre fly (an environmental occupational hazard which is mainly produced during conversion of cotton fibre to fabric). Fibre fly along with cotton dust particles are released into the working place and during yarn preparation, fluffing of cotton is done by holding one end between the teeth and pulling with the other. These fibre enter the mouth and adhere to dental plaque which, in due course of time gets mineralized to form calculus, this formed calculus coupled with their poor oral hygiene practices leads to periodontitis [17, 18- 19]. Thus, with increased age, duration of employment and with decline in education level periodontal health status worsened among textile workers. Therefore, occupational health should be integrated with oral health and occupational health services including oral health should be provided to every worker to improve the oral health of the industrial population.

## Conclusion:

We show that with increased age, duration of employment and with decline in education level periodontal health status worsened among textile industrial compared to biscuit workers. Therefore, a comprehensive oral health promotion programme is highly desirable in the study population, which need to be integrated with their occupational services, so as to lead a good oral health quality of life.

## Figures and Tables

**Table 1:** Distribution of study population according to the type of industry and the age group

**S.no.**	**Industries**	**No. of subjects (%)**	**Age group**				**Mean Age ± SD**	**Range**
			20-30(%)	31-40(%)	41-50(%)	51-60(%)		
1	Biscuit	520 (47.3)	333(64)	154(29.6)	33(6.3)	0(0)	12.29±6.11	(20-48)
2	Textile	580 (52.7)	284(49)	206(35.5)	65(11.2)	25(4.3)	32.27±8.56	(21-59)

**Table 2:** Comparison of CPI and LOA according to adverse oral habits among textile and biscuit industry workers

**S. No.**	**Adverse Habit Category**	**Biscuit Industry (N = 520)**			**Textile Industry (N = 580)**			**Sig. Diff.**	
		**N**	**Mean**	**SD**	**N**	**Mean**	**SD**	**t-value**	**p-value**
**CPI**									
1	None	474	2.44	0.8	396	3.22	0.7	15.856	<0.001
2	Tobacco only	27	2.81	0.8	66	3.65	0.5	6.077	<0.001
3	Alcohol only	14	2.36	0.6	14	3.29	0.5	4.409	<0.001
4	Both	5	2.6	0.9	104	3.56	0.5	3.895	<0.001
	ANOVA (F)	2.173			14.436				
	"p"	0.09			<0.001				
LOA									
1	None	474	0.12	0.6	396	0.5	0.7	8.729	<0.001
2	Tobacco only	27	0.26	0.7	66	1.42	0.7	7.227	<0.001
3	Alcohol only	14	0.29	0.6	14	0.43	0.8	0.55	0.587
4	Both	5	0.4	0.6	104	1.31	0.7	2.708	0.008
	ANOVA (F)	1.325			0.265				
	"p"	55.024			<0.001				

**Table 3:** Multiple Regression showing association of CPI (>3) with duration of employment, type of education and type of industry

**Variable**	**Category**	**CPI >3 n/N (%)**	**Crude OR**	**95% CI**	**Adjusted OR**	**95% CI**	**p-value**
Industry	Biscuit	39/520 (7.5%)	-1.98	-2.86 to -1.10	0.137	0.058-0.323	<0.001
	Textile (Ref)	249/580 (42.9%)	1	-	2.36	-	
Duration of Employment	< 2 years	4/4 (100%)	20.07	20.07-20.07	5 x 10^8^	5 x 10^8^-5 x 10^8^	-
	3-5 years	105/608 (17.3%)	-0.68	-1.16 to -0.20	0.509	0.322-0.807	0.004
	6-10 years	118/360 (32.8%)	-0.004	-0.47 to 0.462	0.996	0.631-1.572	0.988
	>10 years (Ref)	61/128 (47.7%)	1	-	1	-	-
Education	Up to Primary	239/543 (44.0%)	1.303	-1.969 to 2.575	4.354	0.146-12.562	0.789
	Up to Intermediate	48/546 (8.8%)	-0.206	-2.34 to 1.93	2.814	0.100-6.592	0.847
	Graduate & Above (Ref)	1/11 (9.1%)	-0.674	-	1	-	-

**Table 4:** Multiple Regression showing association of LOA with duration of employment, type of education and type of industry

**Variable**	**Category**	**LOA Present n/N (%)**	**Crude OR**	**95% CI**	**Adjusted OR**	**95% CI**	**p-value**
Industry	Biscuit	45/520 (8.7%)	-2.19	-3.05 to -1.33	0.11	0.048-0.258	<0.001
	Textile (Ref)	295/580 (50.9%)	1	-	2.6	-	-
Duration of Employment	< 2 years	4/4 (100%)	16.42	-6504 to 6536	107	0-107	0.996
	3-5 years	111/608 (18.3%)	-1.96	-2.48 to -1.44	0.141	0.085-0.235	<0.001
	6-10 years	129/360 (35.8%)	-1.22	-1.74 to -0.70	0.297	0.178-0.496	<0.001
	>10 years (Ref)	96/128 (75.0%)	1	-	1	-	-
Education	Up to Primary	283/543 (52.1%)	15.45	14.68-16.22	5.12	2.45-8.94	<0.001
	Up to Intermediate	57/546 (10.4%)	11.98	10.98-13.98	3.26	3.22-7.62	<0.001
	Graduate & Above (Ref)	0/11 (0%)	-	-	-	-	-
